# SmBa_1-x_Ca_x_Co_2_O_5+d_ Layered Perovskite Cathodes for Intermediate Temperature-operating Solid Oxide Fuel Cells

**DOI:** 10.3389/fchem.2020.628813

**Published:** 2021-01-25

**Authors:** Kyeong Eun Song, Sung Hun Woo, Seung Wook Baek, Hyunil Kang, Won Seok Choi, Jun Young Park, Jung Hyun Kim

**Affiliations:** ^1^ Department of Advanced Materials Science and Engineering, Hanbat National University, Daejeon, South Korea; ^2^ Interdisciplinary Materials Measurement Institute, Korea Research Institute of Standards and Science (KRISS), Daejeon, South Korea; ^3^ Department of Electrical Engineering, Hanbat National University, Daejeon, South Korea; ^4^ Department of Nanotechnology and Advanced Materials Engineering, HMC, Sejong University, Seoul, South Korea

**Keywords:** intermediate temperature-operating solid oxide fuel cell, cathode, electrical conductivity, area specific resistance, layered perovskite

## Abstract

In SmBa_1-x_Ca_x_Co_2_O_5+d_ (x = 0.01, 0.03, 0.1, and 0.2, SBCCO) oxide systems calcined at 1100°C for 8 h, the XRD patterns of the SBCCO single phase were maintained in the cases of SmBa_0.97_Ca_0.03_Co_2_O_5+d_ (SBCCO-0.97) and SmBa_0.99_Ca_0.01_Co_2_O_5+d_ (SBCCO-0.99) compositions. In SmBa_0.8_Ca_0.2_Co_2_O_5+d_ (SBCCO-0.8) and SmBa_0.9_Ca_0.1_Co_2_O_5+d_ (SBCCO-0.9), CaCoSmO_4_ existed with the pattern SBCCO. SBCCO structures were identified as orthorhombic crystal structures because they showed splitting of the X-ray diffraction (XRD) peaks at 23.4°, 47.9°, and 59.1°.Typical metallic conduction behaviors were found in all measured compositions except SBCCO-0.8, which showed a metal-insulator transition (MIT) behavior. Compared to other SmBa_1-x_Ca_x_Co_2_O_5+d_ compositions, SBCCO-0.8 showed the highest electrical conductivity of 460 S/cm at 500°C. In particular, SBCCO-0.9 was found to have an excellent ASR characteristic of about 0.077 Ωcm^2^ at 700°C. The activation energy of SBCCO-0.9 was the lowest among SBCCO oxide systems with a value of 0.77 eV.

## Research Highlights

SBCCO-0.8: The highest electrical conductivity value of 329.7 S/cm at 700°C.
The lowest ASR value of SBCCO-0.9: 0.077 Ωcm^2^ at 700°C.
The lowest activation energy of SBCCO-0.9: 0.77 eV.
The tendency of ASR was similar to that of unit cell volume.

## Introduction

Solid Oxide Fuel Cells (SOFC) are energy devices that directly convert the chemical energy of hydrogen and oxygen into electrical energy; these devices have higher efficiency than do other energy conversion devices. Significantly, SOFCs operate in a high-temperature range of 650–1000°C. They have many advantages in terms of fuel and material selectivity.

However, the advantages of SOFCs in high temperature conditions may be limited by certain disadvantages: thermal degradation of ceramic materials and metal materials inside SOFCs occurs because of the high temperature conditions (Koide et al., 2000; Andersson et al., 2013; Jin et al., 2017)

To solve these issues, many researchers and institutes have focused on the development of Intermediate Temperature-operating Solid Oxide Fuel Cells (IT-SOFCs), which have lowered operation temperatures.

In particular, research has been conducted on cathode materials that exhibit fast oxygen reduction properties at relatively lower temperatures. The typical cathode material used for IT-SOFCs is a simple perovskite having a chemical composition of ABO_3_ (A: lanthanide, B: transition metal). In addition, a complex perovskite of AA^/^BB^/^O_3_ composition, in which various kinds of elements are substituted for the simple A-site and B-site of perovskite (Complex perovskite) has been found to have excellent electrochemical properties as well as electrical conductivities.

However, complex perovskites can exhibit dis-ordering due to substitution of various materials and decreases of coulomb potential, elastic potential and oxygen mobility (Chen et al., 2003; Hashimoto et al., 2005; Taskin et al., 2007).

To solve these problems, studies of layered perovskites showing chemical composition of AA^/^B_2_O_5+d_ are being conducted. Layered perovskites occupy many vacancies in the oxide lattice, because oxygen ions are partially removed or completely removed in the [Ln-O]_x_ layer. These oxygen vacancies prevent spin glass behavior and enhance the behavior and induce superior 2surface kinetic property (Tarancon et al., 2007; Frontera et al., 2003).

Our research group reported that synthesized layered perovskite, SmBa_0.5_Sr_0.5_Co_2_O_5+d_ (SBSCO), showed an excellent Area Specific Resistance (ASR) of 0.092 Ωcm^2^ at 700°C and could be used as a cathode material of IT-SOFC (Kim et al., 2009b).

However, the Sr contained in SBSCO causes segregation on the surface, causing problems such as reduction of electrochemical properties and long-term performance (Liu et al., 2014; Zhao et al., 2014).

Based on these results, the goal of this study is to investigate the phase synthesis and electrochemical properties of SmBa_1-x_Ca_x_Co_2_O_5+d_ (SBCCO) oxide systems in which elemental Ba and Ca elements were substituted into the A^/^-site of the layered perovskite. Especially, the generation of a secondary phase was investigated in the synthesis process of SBCCO, and not only the effect of the secondary phase on the electrical conductivity, but also its relationship to the crystal structure and electrochemical properties were studied.

## Experimental

### Sample Preparation and X-Ray Diffraction (XRD)

Samarium Oxide (Sm_2_O_3_, 99.9%. Alfa Aesar), Barium Carbonate (BaCO_3_, 99.0%, Alfa Aesar), Calcium Carbonate (CaO, 99.5%, Alfa Aesar), and Cobalt Oxide (Co_3_O_4_, 99.9%, Alfa Aesar) powder were used for the synthesis of SmBa_1-x_Ca_x_Co_2_O_5+d_ (SBCCO, x = 0.01, 0.03, 0.1, and 0.2) by solid state synthesis (SSR). Each powder was accurately weighed according to its chemical composition, and mixed using an agate mortar with a pestle and ethanol. The mixtures were placed in an oven and maintained at 78°C for 12 h to evaporate the ethanol. Mixtures were calcined for 6 h at 1000°C as a first calcination step to decompose all the carbonate. After that, materials were crushed by the agate mortar with the pestle and ball mill; then, a secondary calcination step was carried out for 8 h in an electric furnace at 1100°C in air atmosphere.

The chemical compositions and abbreviations in SmBa_1-x_Ca_x_Co_2_O_5+d_ (SBCCO, x = 0.01, 0.03, 0.1, and 0.2) of cathode materials are summarized in Table 1.

**TABLE 1:** Abbreviations of SmBa_1-x_Ca_x_Co_2_O_5+d_ (SBCCO, x = 0.01, 0.03, 0.1, and 0.2) oxide systems.

Chemical compositions	Abbreviations
SmBa_0.8_Ca_0.2_Co_2_O_5+d_	SBCCO-0.8
SmBa_0.9_Ca_0.1_Co_2_O_5+d_	SBCCO-0.9
SmBa_0.97_Ca_0.03_Co_2_O_5+d_	SBCCO-0.97
SmBa_0.99_Ca_0.01_Co_2_O_5+d_	SBCCO-0.99

X-ray diffraction (XRD) patterns of the synthesized SmBa_1-x_Ca_x_Co_2_O_5+d_ (SBCCO, x = 0.01, 0.03, 0.1, and 0.2) oxide systems were obtained on a Model D/Max 2500, Rigaku (45Kv, 200mA, Cu kα radiation); the obtained data were matched with reference data for the phase synthesis and analyzed using the MDI JADE six program.

### Electrical Conductivity Analysis

To measure the electrical conductivity of the synthesized cathode materials, pellets were prepared by pressing of rectangular-shaped bars (25 × 6 × 3 mm). Then, pellets for the electrical conductivity measurement were sintered at 1100°C for 3 h. The electrical conductivities were measured using the DC four probe method with a Keithley 2400 Source Meter over a temperature range of 50–900°C at steps of 50°C and a heating rate of 5°C/min.

### Electrochemical Characterization

For fabrication of the electrolytes, individual samples of 2.5 g of Ce_0.9_Gd_0.1_O_2_ (CGO91, Rhodia) powder were pressed into disc shaped metal molds at 2 × 10^3^ kg/m^2^. The CGO91 electrolytes were sintered at 1450°C for 6 h and the final geometry of the sintered electrolyte pellets was approximately 22.18 mm in diameter and 0.97 mm in thickness. Inks of single-phase and composite cathodes were prepared using mixtures of SmBa_1-x_Ca_x_Co_2_O_5+d_ (SBCCO) cathode powders, Alpha-terpineol (KANTO CHEMICAL), Butvar (SIGMA Aldrich) and acetone. These mixtures were stirred for 1 week with a magnetic bar. Then, Ce_0.9_Gd_0.1_O_2_ (CGO91, Rhodia) was mixed with the cathode powder at a mass ratio of 1:1 to sustain the decrease in area specific resistance (ASR). After the prepared inks were applied to the electrolytes using screen printing to fabricate symmetrical half cells, samples were heat-treated at 1000°C for 1 h at a heating rate of 5°C/min.

The area specific resistances (ASRs) of the half cells were measured using an AC impedance analyzer (Model nStat, HS Technologies); the measurements were performed in a frequency range of 0.05 Hz–2.5 MHz and temperature range of 900°C–650°C with 50 C steps in air atmosphere.

The cathode ASR was determined from the difference between the first and second intercepts of the impedance curves, divided by 2.

## Results and Discussion

### X-Ray Diffraction (XRD) Analysis of Layered Perovskite Oxide Systems

**FIGURE 1:**
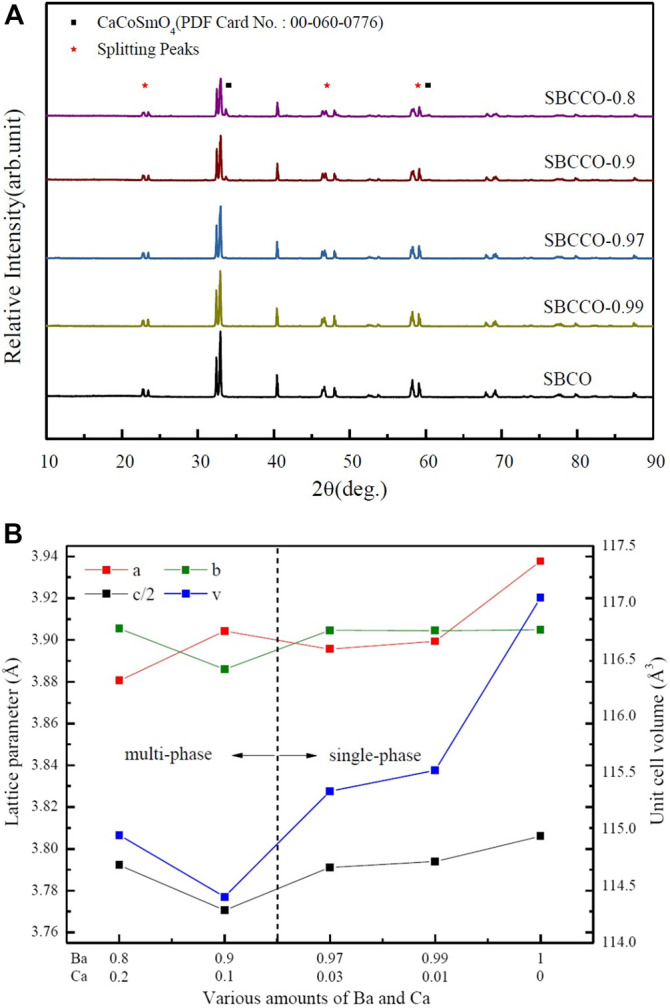
**(A)** X-ray diffraction (XRD) results of SmBaCo_2_O_5+d_ (SBCO) and SmBa_1-x_Ca_x_Co_2_O_5+d_ (SBCCO, x = 0.01, 0.03, 0.1, and 0.2) oxide systems calcined at 1100°C for 8 h under air condition and **(B)** calculated lattice parameters of SBCO and SBCCO oxide systems.

X-ray diffraction (XRD) results of SmBa_1-x_Ca_x_Co_2_O_5+d_ (hereafter SBCCO, x = 0.01, 0.03, 0.1 and 0.2) substituted with Barium (Ba) and Calcium (Ca) in the A^/^-site of the layered perovskite SmBaCo_2_O_5+d_ (SBCO) are shown Figure 1A.

As can be seen in Figure 1A, SmBa_0.97_Ca_0.03_Co_2_O_5+d_ (SBCCO-0.97) and SmBa_0.99_Ca_0.01_Co_2_O_5+d_ (SBCCO-0.99) can be identified as single because typical peaks were found at about 23.4°, 33.0°, 40.4°, 46.4°, 59.1°, 69.2° and 78.0°, which can be considered typical of layered perovskite.

The XRD patterns are the same as those of SmBaCo_2_O_5+d_ (SBCO), reported by our group as a single phase. Therefore, it can be determined that SBCCO-0.97 and SBCCO-0.99 were synthesized as single phase (Kim et al., 2009b).

On the other hand, additional peaks caused by CaCoSmO_4_ (PDF no. 00-060-0776) were measured in the vicinity of 34.0° and 60.3° in the XRD results for SmBa_0.8_Ca_0.2_Co_2_O_5+d_ (SBCCO-0.8) and SmBa_0.9_Ca_0.1_Co_2_O_5+d_ (SBCCO-0.9).

That is, it can be confirmed that the single phase of the layered perovskite and CaCoSmO_4_ coexist in SBCCO-0.8 and SBCCO-0.9.

CaCoSmO_4_ has been reported in the literature to improve the electrical conductivity (Taguchi et al., 2007); the relationship between the appearance of CaCoSmO_4_ and the electrical conductivity will be further explained in the section on Electrical Conductivity Analysis of Layered Perovskite.

In addition, the relative intensity of CaCoSmO_4_ increased as the amount of Ca substitution increased, which means that the concentration of CaCoSmO_4_ increased in SBCCO-0.8 compared to SBCCO-0.9.

Through these results, it is possible to find the composition conditions for synthesis of a single phase depending on the amount of Ca substitution in the composition of SmBa_1-x_Ca_x_Co_2_O_5+d_ (x = 0.01, 0.03, 0.1 and 0.2). The compositions of x = 0.01 and 0.03 in the SmBa_1-x_Ca_x_Co_2_O_5+d_ oxide system appropriate conditions. In other words, single phase SmBa_1-x_Ca_x_Co_2_O_5+d_ oxide systems can be synthesized when elemental Ca which can be substituted at the A^/^-site, exists only in a limited range (x = 0.01 and 0.03).

This can be explained by the difference in the ionic radii of Ba and Ca. When Ca, having a relatively small ionic radius compared to that of Ba, is replaced with SmBa_1-x_Ca_x_Co_2_O_5+d_ oxide systems, a much larger distortion is observed due to the difference of the ionic radii, leading to distortion of the structure of the sublattice. These distortions are not found in SBCO, SBCCO-0.99 or SBCCO-0.97, but can be observed in SBCCO-0.9 and SBCCO-0.8 (Olsson et al., 2017; Wu et al., 2020).

In addition, the peaks measured at 23°, 47° and 59° (2θ) split from the other compositions of SmBa_1-x_Ca_x_Co_2_O_5+d_ (SBCCO, x = 0.01, 0.03, 0.1 and 0.2), as can be seen in Figure 1A. This shows the same behavior as the splitting peaks at 23°, 47° and 59° of SmBa_0.5_Sr_0.5_Co_2_O_5+d_ (SBSCO), SBCO and GdBaCo_2_O_5+d_ (GBCO), previously reported as orthorhombic crystal structures. As a result, the SmBa_1-x_Ca_x_Co_2_O_5+d_ (x = 0.01, 0.03, 0.1, and 0.2) oxide systems were determined to have an orthorhombic crystalline structure with different lattice parameters. (Kim et al., 2009b; Aksenova et al., 2010; Marrero-Jerez et al., 2014).

The calculated lattice parameters and unit cell volumes of the SmBa_1-x_Ca_x_Co_2_O_5+d_ (x = 0.01, 0.03, 0.1 and 0.2) oxide systems obtained using the program JADE six are summarized in Figure 1B, which shows the specific crystallographic characteristics.

For SBCCO-0.97, SBCCO-0.99 and SBCO, considered to be single phase, it was confirmed that the unit cell volumes and lattice parameters decreased as the substitution amount of Ba decreased. This behavior can be explained as stemming from the difference in ionic radii of Ba and Ca substituted into the A^/^-site in the chemical composition of AA^/^B_2_O_5+d_ (A: Lanthanide, A': Ba and Ca): the ionic radius of Ba (1.35 Å) is considerably larger than that of Ca (0.99 Å).

When considering a crystal in which Ba and Ca are substituted into the layered structure, layered perovskite has a structure in which [Ln-O] and [A^/^-O] layers appear along the c-axis. As a result Ca can replace Ba at the A^/^-site when the substitution amount of Ba decreases. As the substitution amount of Ca increases in the SmBa_1-x_Ca_x_Co_2_O_5+d_ (x = 0.01, 0.03, 0.1, and 0.2) oxide systems, the distance between the [Ln-O] layer and the [A^/^-O] layer decreases; these relationships result in decreased lattice parameters and, accordingly, the unit cell volume also decreases (Ahrens, 1952; Pauling, 1931; Kim and Manthiram, 2015).

### Electrical Conductivity Analysis of Layered Perovskite

**FIGURE 2:**
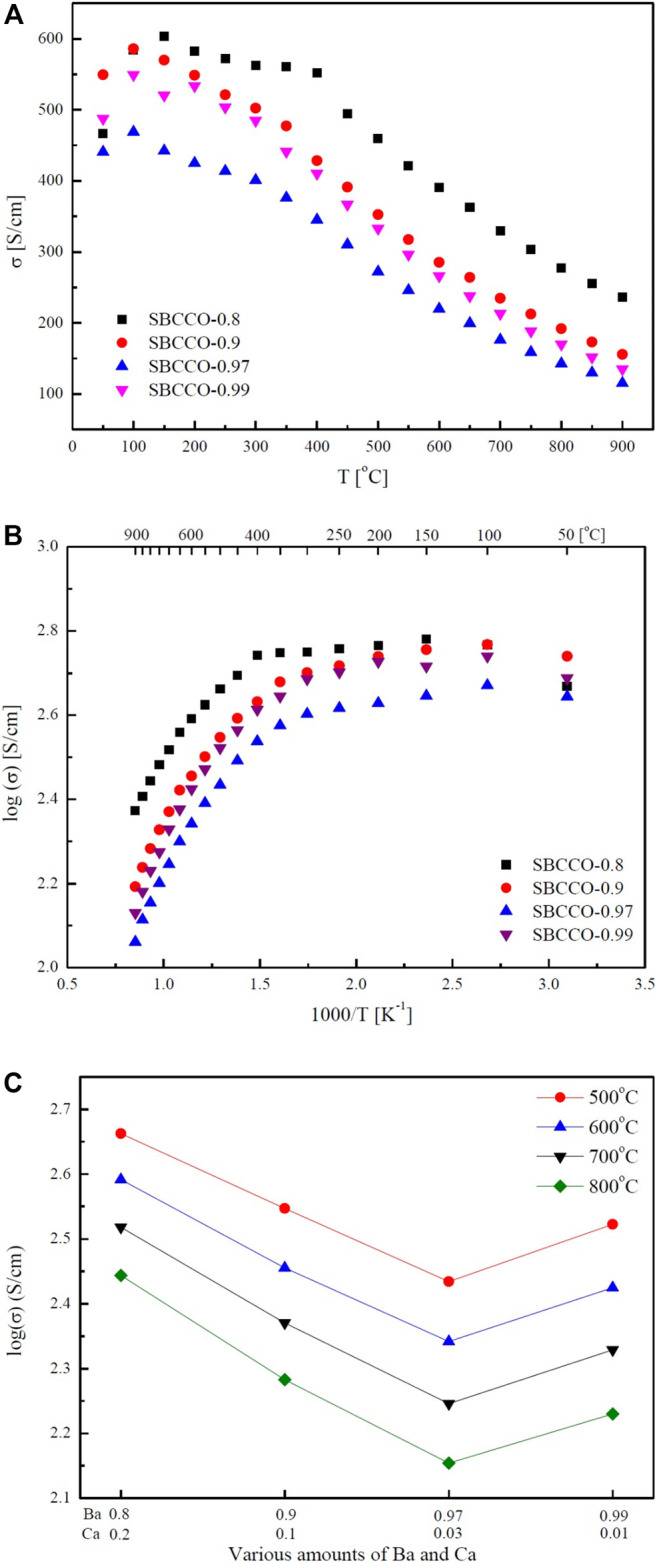
Electrical conductivities of SmBa_1-x_Ca_x_Co_2_O_5+d_ (SBCCO, x = 0.01, 0.03, 0.1, and 0.2) Layered perovskite oxide systems with respect to the temperature: **(A)** electrical conductivity vs. T (^o^C), **(B)** logarithm of electrical conductivity vs. 1000/T (K^−1^), and **(C)** electrical conductivity results with respect to various amounts of Ba (Barium) and Ca (Calcium).

Electrical conductivity results of SmBa_1-x_Ca_x_Co_2_O_5+d_ (x = 0.01, 0.03, 0.1 and 0.2) oxide systems with respect to the composition and temperature are summarized in Figure 2. From Figure 2A, the maximum electrical conductivities in these materials can be observed to be in the lower temperature ranges; the minimum values are measured in the higher temperature ranges. The maximum and minimum electrical conductivity values with respect to the temperature are summarized in Table 2.

**TABLE 2:** Maximum and minimum conductivities and temperatures of SmBa_1-x_Ca_x_Co_2_O_5+d_ (SBCCO, x = 0.01, 0.03, 0.1, and 0.2).

Chemical compositions	Maximum conductivity (S/cm)	Temperature(°C)	Minimum conductivity (S/cm)	Temperature(°C)
SBCCO-0.8	603	150	236	900
SBCCO-0.9	586	100	156	900
SBCCO-0.97	469	100	115	900
SBCCO-0.99	549	100	135	900

According to Figure 2A and Table 2, the maximum electrical conductivity value (603 S/cm) of SBCCO-0.8 is the highest of all compositions tested and the maximum electrical conductivity value decreases when the substitution amount of Ca decreases in SmBa_1-x_Ca_x_Co_2_O_5+d_ (x = 0.01, 0.03, 0.1 and 0.2) oxide systems. In addition, SBCCO-0.8 shows its maximum electrical conductivity value at 150°C, but other compositions show maximum conductivity at 100°C.

The value of electrical conductivity in SmBa_0.5_Sr_0.5_Co_2_O_5+d_ (SBSCO), at about 1280 S/cm at 50°C, is better than the values of SmBaCo_2_O_5+d_ (SBCO, 500 S/cm) and SBCCO-0.8 (466 S/cm). However, the value of electrical conductivity in SBCCO-0.8 was 330 S/cm at 700°C, which was lower than that of SBSCO (430 S/cm) but higher than that of SBCO (270 S/cm) (Kim et al., 2010; Kim, 2014 ).

The overall electrical conductivity behaviors can be identified in Figure 2B, which shows the conductivity results at logarithmic scale; the SmBa_1-x_Ca_x_Co_2_O_5+d_ oxide systems show typical metallic behavior, in which the electrical conductivity decreases with increasing temperature, except for the SmBa_0.8_Ca_0.2_Co_2_O_5+d_ (SBCCO-0.8) composition (Kim et al., 2010).

All of the compositions used in this conductivity measurement showed relatively higher electrical conductivity values from relatively lower temperature ranges (50–300°C) and a rapid decrease from 300 to 900°C, which implies that metal–insulator transition (MIT) behavior is observed in SmBa_1-x_Ca_x_Co_2_O_5+d_ (x = 0.01, 0.03, 0.1, and 0.2) oxide systems (Kim, 2014).

SBCCO-0.8 showed the same MIT behavior; however, the temperature at which the maximum conductivity value is measured is relatively lower compared to the other compositions; the electrical conductivity increased in the 50–150°C range and decreased at temperatures above 200°C in SBCCO-0.8.

The electrical conductivity is relatively high in the low temperature range in the composition of SmBa_1-x_Ca_x_Co_2_O_5+d_ (SBCCO, x = 0.01, 0.03, 0.1 and 0.2), as shown in Figure 2B. This is the concentration of Co^4+^ that causes small polaron hopping, which is relatively higher than the case for Co^3+^, in the state where Co^3+^ and Co^4+^ coexist (Kim et al., 2010). The effect of these Co^4+^ concentrations on the electrical conductivity in the low temperature range was reported in our group (Kim et al., 2010).

On the other hand, the decreased electrical conductivity values in the range of 300–900°C are caused by the decreased concentration of Co^4+^. Further, the concentration of oxygen vacancies increases as a function of temperature in these oxide systems. This can also result in decreases in electrical conductivity. For example, the movement of charge carriers can be limited due to oxygen vacancies, which increase rapidly in the range of 300–350°C; at the same time, the electrical conductivity decreases rapidly (Kim, 2014). It can be seen in the literature results of Thermogravimetric Analysis (TGA) and Differential Scanning Calorimetry (DSC) of SmBa_0.5_Sr_0.5_Co_2_O_5+d_ (SBSCO) that weight decreases rapidly from 300 to 350°C; the same tendency was found for the electrical conductivity in this study (Kim, 2014).

The results of the electrical conductivity according to the composition of SmBa_1-x_Ca_x_Co_2_O_5+d_ (SBCCO, x = 0.01, 0.03, 0.1 and 0.2) oxide systems from 500 to 700°C are presented in Figure 2C. The electrical conductivity of SBCCO-0.8 was the highest at the measured temperature and composition. The value of electrical conductivity in SBCCO-0.8 is highest, at about 460 S/cm, from 500°C, better than the values of SmBa_0.9_Ca_0.1_Co_2_O_5+d_ (SBCCO-0.9, 352 S/cm), SmBa_0.97_Ca_0.03_Co_2_O_5+d_ (SBCCO-0.97, 272 S/cm) and SmBa_0.99_Ca_0.01_Co_2_O_5+d_ (SBCCO-0.99, 333 S/cm). In other words, the compositions in which the single phase and CaCoSmO_4_ (PDF no. 00-060-0776) coexist generally have higher electrical conductivity than the compositions of single phase. Therefore, it can be considered that this result was affected by the increase in concentration of CaCoSmO_4_.

According to the literature, CaCoSmO_4_ improves the electrical conductivity as the temperature increases. This effect can also be confirmed for SBCCO-0.8, which contains the most CaCoSmO_4_, and has the highest electrical conductivity (Taguchi et al., 2007).

In addition, it can be seen that the electrical conductivity of SBCCO-0.97, which has the lowest value of electrical conductivity among all compositions, is higher than 100 S/cm, which is the minimum electrical conductivity required for IT-SOFC (Boehm et al., 2003). Therefore, all compositions of SmBa_1-x_Ca_x_Co_2_O_5+d_ (SBCCO, x = 0.01, 0.03, 0.1 and 0.2) are applicable as cathode material of IT-SOFC.

### Electrochemical Characterization

**FIGURE 3:**
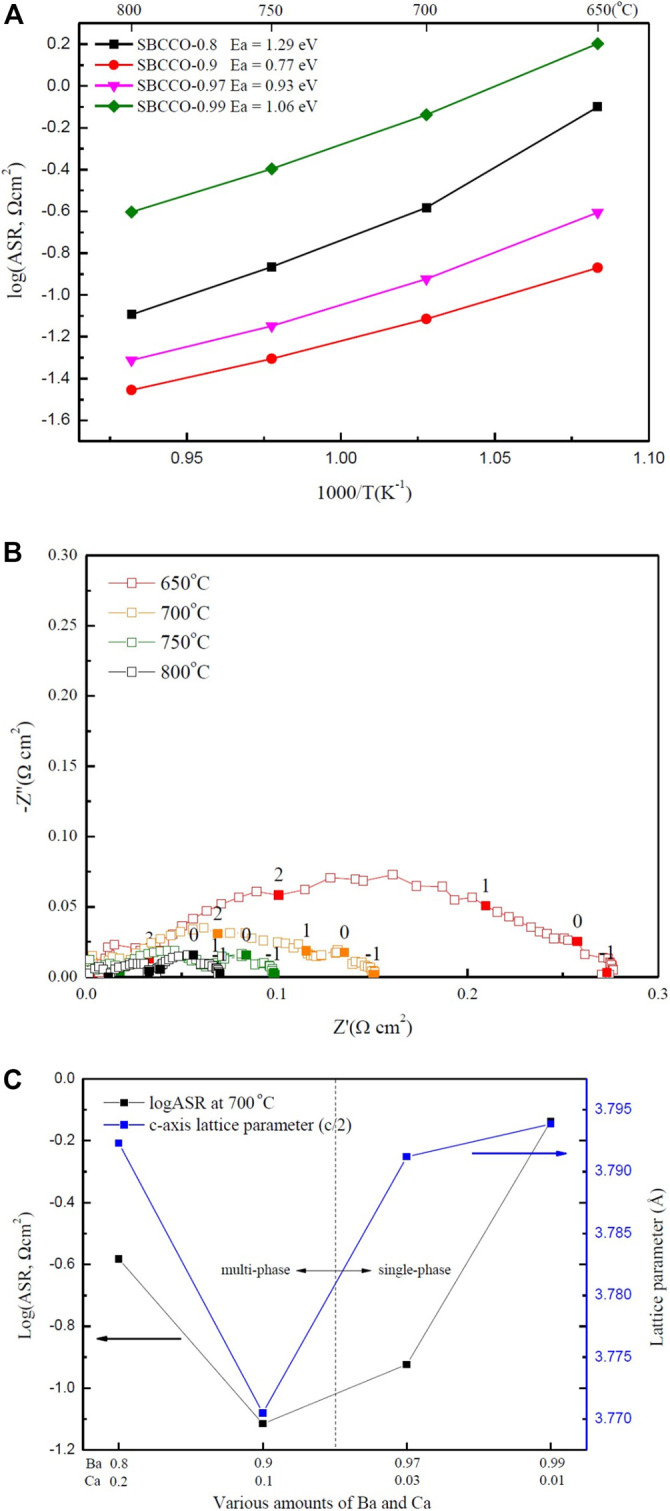
**(A)** Area specific resistances (ASRs) of SmBa_1-x_Ca_x_Co_2_O_5+d_ (SBCCO, x = 0.01, 0.03, 0.1, and 0.2) oxide systems measured from 650 to 800°C in air condition, **(B)** Impedance plots of SBCCO-0.9 oxide system measured at 650, 700, 750, and 800°C in air on dense CGO91 electrolyte and **(C)** Relationships between ASRs measured at 700°C and calculated c-axis lattice parameters of SBCCO oxide systems.

To investigate the area specific resistances (ASRs) of SmBa_1-x_Ca_x_Co_2_O_5+d_ (SBCCO, x = 0.01, 0.03, 0.1 and 0.2), impedance spectroscopy was carried out using symmetrical half cells in a temperature range of 650–850°C; the results are shown in Figure 3A.

ASR was compared by considering the surface area of the cathode after sintering at high temperature. Since all half cells are screen printed with the same size mesh, there is little difference in surface area.

The results of ASR have the ohmic resistance removed to allow comparison with the ASR of SBCCO. The ASR values ​​of SBCCO-0.97 and SBCCO-0.99 were 0.11 Ωcm^2^ and 0.72 Ωcm^2^ at 700°C. The ASRs ​​of SBCCO-0.8 and SBCCO-0.9 were 0.26 Ωcm^2^ and 0.07 Ωcm^2^ at 700°C. The SBCCO-0.9 cathode material showed the lowest ASR value at 700°C. In addition, the ASR of SBCCO-0.9 was found to be 0.12 Ωcm^2^ at 650°C; this is the lowest ASR value among the oxide systems used in this experiment.

When comparing the ASR value (0.12 Ωcm^2^) of SBCCO-0.9 with the 0.244 Ωcm^2^ value of SmBa_0.5_Sr_0.5_Co_2_O_5+d_ (SBSCO), 0.13 Ωcm^2^ value of SmBaCo_2_O_5+d_ (SBCO) and 0.558 Ωcm^2^ value of GdBa_0.5_Sr_0.5_Co_2_O_5+d_ (GBSCO) at 650°C, SBSCO-0.9 showed the lowest ASR value, which indicates that the oxygen reduction processes in Ca-substituted SBSCO-0.9 occurs faster than in Sr-substituted layered perovskites (Kim et al., 2009a; Kim et al., 2009b; Song et al., 2018).

The activation energies of SmBa_1-x_Ca_x_Co_2_O_5+d_ (SBCCO, x = 0.01, 0.03, 0.1 and 0.2) oxide systems were calculated from Arrhenius plots of the fitted line and their values are also summarized in Figure 3A. SBSCO-0.9 showed the lowest activation energy (0.77 eV) and SBSCO-0.97 showed a relatively low activation energy of 0.93 eV.

However, the activation energies of SBCCO-0.99 and SBCCO-0.8 are 1.06 and 1.29 eV; these values are higher than those of SBSCO-0.9 and SBSCO-0.97. It can be seen that SBCCO-0.9 exhibits lower activation energy even when compared with the activation energy of the Sr-substituted layered perovskites because SBSCO, SBCO and GBSCO have activation energies of 1.23, 1.11 and 1.22 eV. This means that the energy to activate the reactions for SBCCO-0.9 is smaller than those for SBSCO, SBCO and GBSCO (Kim et al., 2009a; Kim et al., 2009b; Song et al., 2018 )

The activation energies of Sr-substituted perovskite La_0.6_Sr_0.4_Co_0.2_Fe_0.8_O_3_ (LSCF) and Ba_0.5_Sr_0.5_Co_0.2_Fe_0.8_O_3_ (BSCF) are 0.44 and 0.38 eV, lower than that of SBCCO-0.9 (Shen and Lu, 2018). However, SBCCO-0.9 has the advantage of compatibility with lower case Yttria-stabilized zirconia (YSZ) electrolyte without interlayer. The cathode substituted Sr uses an interlayer between the cathode and YSZ electrolyte to prevent segregation (Cai et al., 2012; Wang et al., 2016 ). SBCCO substitutes Ca instead of Sr, so it will be compatible with YSZ electrolyte without interlayer.

Figure 3B shows ASR results of SBCCO-0.9. The composition of SBCCO-0.9 has ASR values of 0.13, 0.07, 0.04 and 0.03 Ωcm^2^ at 650, 700, 750 and 800°C. These values are all lower than 0.15 Ωcm^2^, which is the ASR required of cathodes of IT-SOFC at 650°C (Steele, 1996).

Figure 3C shows the correlation between the lattice parameters and the ASRs. The SBCCO-0.9 composition showed the lowest ASR, with the lowest lattice parameter on the c-axis. Therefore, the decrease in ASR was affected by the decrease in c-axis lattice parameter due to the increase in amount of Ca. The SBCCO-0.9 composition comprised of multi phases shows the lowest ASR property among all SmBa_1-x_Ca_x_Co_2_O_5+d_ (SBCCO, x = 0.01, 0.03, 0.1, and 0.2) layered perovskites and is directly related with the crystallographic properties caused by the decrease of the c-axis lattice parameters.

## Conclusion

In this research, we have investigated the phase synthesis and electrochemical properties of SmBa_1-x_Ca_x_Co_2_O_5+d_ (SBCCO, x = 0.01, 0.03, 0.1 and 0.2) layered perovskites by substituting Ca for Ba as possible cathode material for IT-SOFC.

The XRD results of the SmBa_1-x_Ca_x_Co_2_O_5+d_ oxide system reveal that single phases were found in the compositions of SBCCO-0.97 (x = 0.03) and SBCCO-0.99 (x = 0.01). However, compositions such as SBCCO-0.8 and SBCCO-0.9 included secondary phases of CaCoSmO_4_. The electrical conductivities of SBCCO-0.8 and SBCCO-0.9 were about 460 S/m and 352 S/cm at 500°C; these values are superior to those of the single-phase cathode. In addition, SBCCO-0.9 has excellent ASR values of 0.13 and 0.07 Ωcm^2^ at 650 and 700°C.

## Data Availability

The original contributions presented in the study are included in the article/Supplementary Material, further inquiries can be directed to the corresponding author.
